# The Fermionic Massless Modular Hamiltonian

**DOI:** 10.1007/s00220-025-05253-1

**Published:** 2025-03-05

**Authors:** Francesca La Piana, Gerardo Morsella

**Affiliations:** 1 Department of Mathematics, University of Oslo, P.O. Box 1053, 0316 Blindern, Oslo, Norway; 2 Dipartimento di Matematica, Università di Roma Tor Vergata, Via della Ricerca Scientifica, 1, 00133 Rome, Italy

## Abstract

We provide an explicit expression for the modular hamiltonian of the von Neumann algebras associated to the unit double cone for the (fermionic) quantum field theories of the 2-component Weyl (helicity 1/2) field, and of the 4-component massless Dirac and Majorana fields. To this end, we represent the one particle spaces of these theories in terms of solutions of the corresponding wave equations, and obtain the action of the modular group on them. As an application, we compute the relative entropy between the vacuum of the massless Majorana field and one particle states associated to waves with Cauchy data localized in the spatial unit ball.

## Introduction

A milestone of the theory of operator algebras, the Tomita-Takesaki modular theory associates a canonical one parameter group of automorphisms $\{ \sigma ^\varphi _t\}_{t \in {{\mathbb {R}}}}$, the *modular group*, to every von Neumann algebra $\mathscr {M}$ equipped with a faithful normal state $\varphi$ [21]. The *modular Hamiltonian* is then the self-adjoint generator $\log \Delta _\varphi$ of the unitary group implementing the modular group in the GNS representation $\pi _\varphi$ of $\varphi$:

$$\begin{aligned} \Delta _\varphi ^{it} \pi _\varphi (A) \Delta _\varphi ^{-it} = \pi _\varphi (\sigma ^\varphi _t(A)), \qquad A \in \mathscr {M},\,t \in {{\mathbb {R}}}. \end{aligned}$$

In the algebraic approach to quantum field theory [15], for every (open) region *O* of Minkowski space time ${\mathbb {R}}^4$, we consider the von Neumann algebra $\mathscr {A}(O)$ generated by all the observables that are measurable in that region, or, more generally, the (larger) von Neumann algebra $\mathscr {F}(O)$ generated by all the (charged) fields localized in *O*. Under standard assumptions on the net $O \mapsto \mathscr {F}(O)$, (resp. $O \mapsto \mathscr {A}(O)$) the Reeh-Schlieder theorem states that, if both *O* and its causal complement $O'$ have non empty interiors, the vacuum vector $\Omega$ is cyclic and separating for each algebra $\mathscr {F}(O)$ (resp. $\mathscr {A}(O)$), so the restriction to this algebra of the vacuum state $\omega = \langle \Omega , (\cdot )\Omega \rangle$ is normal and faithful, and we can consider the associated modular Hamiltonian $\log \Delta _O$.

It is then natural to try to give an explicit description of the modular Hamiltonian for a certain region of Minkowski space in a given field theory. Apart from its intrinsic interest, which dates back to the very discovery of modular theory at the end of the 60 s, this problem has attracted renewed attention due to the connection between the modular Hamiltonian and Araki’s relative entropy [1], and to the interest in the computation of information theoretic quantities in quantum field theory, which has been rapidly developing in the last decades (see, e.g., [2, 7–10, 18, 23] and references therein).

We have some geometric examples of determination of the modular Hamiltonian. Bisognano and Wichmann [3, 4] described the modular operators for a wedge region in the representation of the vacuum in a model independent setting, starting from the observation that a certain Lorentz boost leaves the wedge invariant; the modular group is indeed induced by this boost. Then Buchholz [6] showed that the modular group of the future light cone, for massless free field theory in odd spatial dimensions $>1$, is induced by the group of global dilatations. Building on these results, and exploiting the geometric relationship between the unit double cone centered at the origin $O_1$ and the wedge, provided by the conformal symmetry of the theory, Hislop and Longo [16] obtained the modular group associated with $O_1$ in the case of the massless free scalar field, in terms of a one parameter group of conformal symmetries preserving the double cone. These results have been generalised to the case of massless free fields with non-zero helicity in [17].

Analogously to what has been done in the scalar field case in [19], in the present work we employ the results of [17] to compute the one particle modular Hamiltonian of the field algebra associated to the unit double cone for the massless Weyl (i.e., helicity $\frac{1}{2}$), Dirac and Majorana fields. For any of these theories, the modular Hamiltonian on the corresponding fermionic Fock space is then the second quantization of the one particle one. The action of the modular Hamiltonian on the (smooth, compactly supported) Cauchy data $\Psi _0: {{\mathbb {R}}}^3 \rightarrow {{\mathbb {C}}}^4$ of the Dirac equation is given by

$$\begin{aligned} i \log \Delta _{O_1}\Psi _0({\varvec{x}}) = -\pi \big [(1-r^2) \partial _k - x_k\big ] \gamma ^0\gamma ^k \Psi _0({\varvec{x}}), \end{aligned}$$

where $\gamma ^0$ and $\gamma ^k$ ($k=1,2,3$) are the Minkowski gamma matrices (see (4.2)) and $r= \left| {\varvec{x}}\right|$, ${\varvec{x}}\in {{\mathbb {R}}}^3$.

Our motivation for working with the Cauchy data of solutions of the Weyl or Dirac equations is that we hope that this setting can be of some use in the much more difficult open problem of computing the double cone modular Hamiltonian for the corresponding massive theories. This is suggested by the fact that, due to the canonical anticommutation relations, the C*-algebra (and possibly the von Neumann algebra) generated by the Cauchy data of the Dirac field is mass independent. It is also interesting to remark that, conversely, in the scalar case detailed knowledge of $\log \Delta _{O_1}$ seems fundamental in order to obtain a direct proof of the mass independence of the algebra $\mathscr {A}(O_1)$ [11].

As an application of the above result, we compute, in the massless Majorana field case, the relative entropy between the restrictions to $O_1$ of the vacuum and of the one particle state $\omega _\Psi$ induced by a Majorana solution $\Psi$ of the Dirac equation with Cauchy data supported in the unit ball, obtaining

$$\begin{aligned} S(\omega _\Psi \Vert \omega ) =\frac{1}{8\pi ^2} \int _{{{\mathbb {R}}}^3} d{\varvec{x}}\,(1-r^2) \langle \Psi , T_{00}(0,{\varvec{x}}) \Psi \rangle , \end{aligned}$$

with

$$\begin{aligned} T_{00}(x) = \frac{i}{2}: \psi ^\dagger (x) \partial _0 \psi (x) - \partial _0 \psi ^\dagger (x) \psi (x): \end{aligned}$$

the quantum energy density of the Majorana field $\psi$ (see the beginning of Sect. 2 for our notations on spinorial Wightman fields).

The above formula for the relative entropy is to be compared with the analogous one obtained in [19], where the canonical energy density of the massless Klein-Gordon field appears together with an extra term proportional to the field’s square, and suggests that the traceless stress-energy tensor should appear there too (see also [22]). This is also coherent with the expression of $\log \Delta _{O_1}$ in terms of the generator of special conformal transformations and with the classical Noether theorem. A general discussion of relative entropy for CAR algebras has recently appeared in [13].

The rest of the paper in organized as follows. In Sect. 2, for the paper to be reasonably self-contained, we briefly review the main results of [17], and we compute the action of the modular group of $O_1$ on certain one particle vectors of the form $\phi (f)\Omega$, $\phi$ the Weyl field. In Sect. 3, we provide the description of the one particle Hilbert space of the Weyl field in terms of (Cauchy data of) solutions of the Weyl equation, called waves, using the correspondence between the test functions and the solutions obtained by convolution with the causal propagator; then we express the action of the modular group on the wave space, and compute the modular Hamiltonian. Finally, in Sect. 4, we use the decomposition of the 4-component Dirac and Majorana fields in terms of 2-components Weyl fields to obtain the massless Dirac and Majorana fields’ local modular Hamiltonians, which is then in turn used to compute the relative entropy $S(\omega _\Psi \Vert \omega )$. The Appendices A and B contain some technical facts used in the main text.

Part of this work is the subject of the first author’s Master thesis in Mathematics, University of Roma Tor Vergata (2022), done under the supervision of the second author.

## Weyl Field Modular Group in the Spacetime Formulation

In this section we compute an explicit formula for the action of the modular group on the one particle vectors of the free helicity 1/2 field in the spacetime formulation, which is not provided in [17] and which we will need later on to obtain the modular hamiltonian in the Cauchy data formulation.

We start by introducing some notation. We will only use lower indices to denote the components of 3-vectors ${\varvec{x}}= (x_1,x_2,x_3) \in {{\mathbb {R}}}^3$ and of 4-vectors $x = (x_0, {\varvec{x}}) \in {{\mathbb {R}}}^4$. Accordingly, we will set $\partial _\mu := \frac{\partial }{\partial x_\mu }$, $\mu =0,\ldots , 3$. Unless otherwise stated, we employ the summation convention over repeated indices. As usual, ${\varvec{p}}\cdot {\varvec{x}}= p_j x_j$ and $px = p_0 x_0 - {\varvec{p}}\cdot {\varvec{x}}$ are the euclidean and Minkowski scalar product of the 3-vectors ${\varvec{p}}, {\varvec{x}}\in {{\mathbb {R}}}^3$ and of the 4-vectors $p=(p_0,{\varvec{p}}), x = (x_0, {\varvec{x}}) \in {{\mathbb {R}}}^4$ respectively. We indicate by  and $C^\infty _c(O,{{\mathbb {C}}}^s)$, $s \in {{\mathbb {N}}}$, respectively the spaces of Schwartz and smooth, compactly supported functions with values in ${{\mathbb {C}}}^s$ and support contained in the open set $O \subset {{\mathbb {R}}}^d$ ($d=3,4$). In particular, if *O* is bounded, then  and $C^\infty _c(O,{{\mathbb {C}}}^s)$ coincide. We use the following conventions for the Fourier transforms of Schwartz functions on ${{\mathbb {R}}}^4$ and ${{\mathbb {R}}}^3$ respectively:

$$\begin{aligned} {\hat{f}}(p):= \int _{{{\mathbb {R}}}^4} dx\,f(x) e^{i px}, \quad p \in {{\mathbb {R}}}^4, \qquad {\hat{g}}({\varvec{p}}):= \int _{{{\mathbb {R}}}^3} d{\varvec{x}}\,g({\varvec{x}}) e^{-i {\varvec{p}}\cdot {\varvec{x}}}, \quad {\varvec{p}}\in {{\mathbb {R}}}^3. \end{aligned}$$

The Fourier transform of vector valued functions is defined componentwise.

Elements $\Psi \in {{\mathbb {C}}}^s$, called spinors, will be thought of as column matrices, and their indices will be denoted by greek letters. The standard norm on ${{\mathbb {C}}}^s$ is denoted by $|\cdot |$. Matrix notation will be used whenever it does not cause confusion. In particular, $A^t$, $A^\dagger$ denote respectively the transpose and the hermitian conjugate of the matrix *A*. Similar notations will be used for spinorial Wightman fields (operator valued distributions): i.e., given a multiplet of Wightman fields $\psi _\alpha$, $\alpha = 1,\ldots , s$, we will define $\psi (f):= \psi _1(f_1) + \cdots + \psi _s(f_s)$, , and we will denote by $\psi (x)$ the column matrix of operator valued distributions with entries $\psi _\alpha (x)$, $\alpha = 1,\ldots ,s$, so that the formal equation

$$\begin{aligned} \psi (f) = \int _{{{\mathbb {R}}}^4} dx f(x)^t \psi (x) \end{aligned}$$

holds. As a consequence, $\psi ^\dagger (x)$ will be the row matrix with entries $\psi _\alpha ^*(x)$, $\alpha = 1,\ldots ,s$, the Wightman fields defined as usual by $\psi _\alpha ^*(f):= \psi _\alpha ({\bar{f}})^*$, . So that, e.g., given an $s \times s$ complex matrix *A*, we will have

$$\begin{aligned} \psi ^\dagger (x) A \psi (y) = \psi ^*_\alpha (x) A_{\alpha \beta }\psi _\beta (y) \end{aligned}$$

as an operator valued distribution on ${{\mathbb {R}}}^4\times {{\mathbb {R}}}^4$.

As customary, we denote by $\sigma _0 = \mathbb {1}$ the $2 \times 2$ identity matrix, and by

$$\begin{aligned} \sigma _1 = \left[ \begin{matrix}0 & \quad 1 \\ 1 & \quad 0\end{matrix}\right] , \quad \sigma _2 = \left[ \begin{matrix}0 & \quad -i \\ i & \quad 0\end{matrix}\right] , \quad \sigma _3 = \left[ \begin{matrix}1 & \quad 0 \\ 0 & \quad -1\end{matrix}\right] , \end{aligned}$$

the three Pauli matrices. To a 4-vector $x = (x_0, {\varvec{x}}) \in {{\mathbb {R}}}^4$ we associate the hermitian matricesfor which  (Minkowski square).

For the convenience of the reader, we now briefly summarize the main results of [17] (to which we refer for proofs and further details) which we will need in the following.

The free, helicity 1/2, right-handed field on 4-dimensional Minkowski spacetime is given by a pair of (bounded) Wightman fields $\phi _\alpha$, $\alpha = 1,2$, acting on the fermionic Fock space  on the one particle Hilbert space . These fields satisfy in the distributional sense the right-handed Weyl equationand canonical anticommutation relations (CAR)

2.1 $$\begin{aligned} \{ \phi _\alpha (x), \phi ^*_\beta (y)\} = S_{\alpha \beta }(x-y), \end{aligned}$$

where $\{\cdot ,\cdot \}$ denotes the anticommutator, and $S:= \tilde{\partial }D$, with *D* the commutator (Pauli-Jordan) distribution of the Klein-Gordon field

$$\begin{aligned} D(x) = \frac{i}{(2\pi )^3}\int _{{{\mathbb {R}}}^4} dp\,\varepsilon (p_0)\delta (p^2)e^{-ipx}. \end{aligned}$$

The associated twisted local net of von Neumann algebras is defined bywith $O \subset {{\mathbb {R}}}^4$ open, and $\phi (f):= \phi _1(f_1)+\phi _2(f_2)$. There is also on  a unitary, strongly continuous representation *U* of the group *SU*(2, 2) of all complex $4 \times 4$ matrices *g* such that

$$\begin{aligned} g B g^\dagger = B, \quad \det g = 1, \qquad \text {with }B = \left[ \begin{matrix} 0 & -i \\ i & 0\end{matrix}\right] (2 \times 2\, \text {blocks)}, \end{aligned}$$

which is a four-fold cover of the 4-dimensional conformal group. The group *SU*(2, 2) acts on the tube $T:= {{\mathbb {R}}}^4+i V_+$ by $(g,z) \in SU(2,2) \times T \mapsto gz \in T$, where2.2Moreover, the universal cover $SL(2,{{\mathbb {C}}}) < imes {{\mathbb {R}}}^4$ of the Poincaré group is identified with the subgroup of *SU*(2, 2) of the matricesThen, the restriction to this subgroup of (the limit as ${\textrm{Im}\,}z \rightarrow 0$ of) the action (2.2) is the usual action of $SL(2,{{\mathbb {C}}}) < imes {{\mathbb {R}}}^4$ on Minkowski space, and the net $O \mapsto \mathscr {F}(O)$ is covariant with respect to the restriction of the representation *U* to $SL(2,{{\mathbb {C}}}) < imes {{\mathbb {R}}}^4$. Furthermore, the representation of the translations $y \in {{\mathbb {R}}}^4 \mapsto U(p(1,y))$ has spectrum contained in ${\bar{V}}_+$ (spectrum condition).

The action of *SU*(2, 2) is most easily described thanks to the existence of strongly analytic (vector valued) functions , , $\alpha = 1,2$, whose boundary value for ${\textrm{Im}\,}z \rightarrow 0$ are the vector valued distributions $\phi _\alpha (x)\Omega$, $\phi _\alpha ^*(x)\Omega$ [17, Thm. 3.4], i.e., such that, for all ,

2.3 $$\begin{aligned} \phi (f)\Omega= &   \lim _{\begin{array}{c} y \rightarrow 0\\ y \in V_+ \end{array}} \int _{{{\mathbb {R}}}^4} dx f(x)^t \phi (x+iy)\Omega , \nonumber \\ \phi (f)^*\Omega= &   \lim _{\begin{array}{c} y \rightarrow 0\\ y \in V_+ \end{array}} \int _{{{\mathbb {R}}}^4} dx f(x)^\dagger \phi ^*(x+iy)\Omega . \end{aligned}$$

Indeed, the representation *U* acts on these functions as [17, Thm. 3.10, Rem. 3.11]2.4Moreover, thanks to the spectrum condition, the translation representation can be extended to the tube *T*, obtaining a strongly continuous semi-group of bounded operators *U*(*p*(1, *w*)), $w \in T$, such that $U(p(1,w))\phi (z)\Omega = \phi (z+w)\Omega$ for all $z,w \in T$.

The modular group of the von Neumann algebra associated to the unit double cone $O_1 = \{ x \in {{\mathbb {R}}}^4:\,|x_0|+|{\varvec{x}}| < 1\}$, with the vacuum $\Omega$ as standard vector, is then expressed in terms of the *SU*(2, 2) representation *U* by [17, Thm. 4.8]

2.5 $$\begin{aligned} \Delta _{O_1}^{i\lambda } = U(e(2\pi \lambda )), \qquad e(\lambda ) = \left[ \begin{matrix} \cosh \frac{\lambda }{2} &  -\sinh \frac{\lambda }{2}\\ -\sinh \frac{\lambda }{2} & \cosh \frac{\lambda }{2}\end{matrix}\right] (2 \times 2\, \text {blocks)}, \quad \lambda \in {{\mathbb {R}}}.\quad \end{aligned}$$

The one parameter subgroup $e(\lambda )$ of *SU*(2, 2) acts on $z \in T$, through (2.2), aswith

2.6 $$\begin{aligned} \tau (\lambda , z) = \frac{1}{2}(1+\cosh \lambda )-\frac{1}{2} z^2(1-\cosh \lambda ) -z_0 \sinh \lambda . \end{aligned}$$

Extending $\nu _\lambda$ to the boundary ${{\mathbb {R}}}^4$ of *T* one gets a (singular) map

**Fig. 1:**
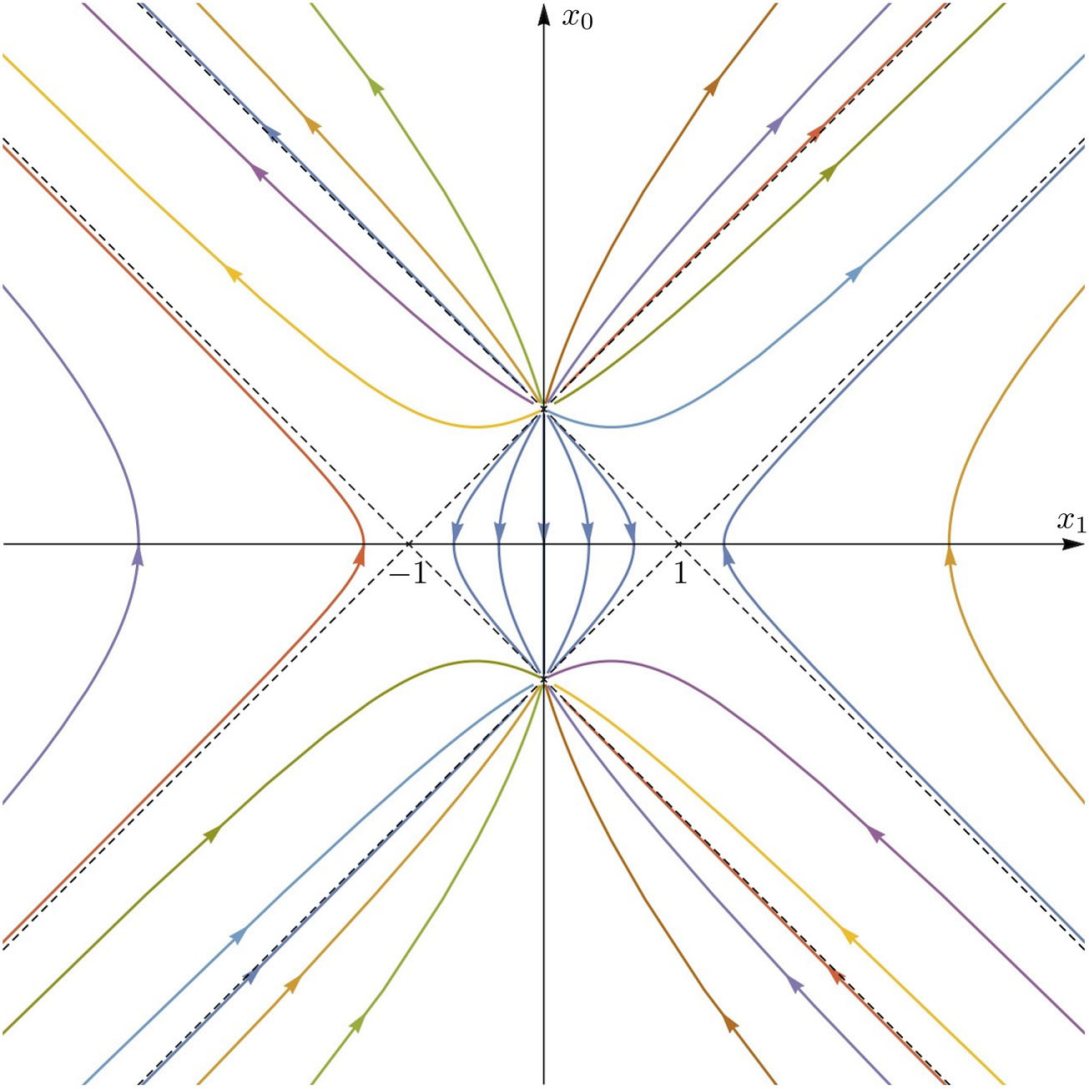
The $O_1$ modular flow on the $x_0, x_1$ plane. The trajectories are continuous on the conformal compactification of Minkowski space. Those outside $O_1$ are singular on Minkowski, and their different branches share the same color

2.7 $$\begin{aligned} \nu _\lambda \!:\! {{\mathbb {R}}}^4 \setminus S_\lambda \rightarrow {{\mathbb {R}}}^4, \,\, x\!=\!(x_0,{\varvec{x}}) \mapsto \nu _\lambda (x) \!=\!\frac{1}{\tau (\lambda ,x)}\left( x_0 \cosh \lambda \!-\! \frac{1}{2} (1\!+\!x^2) \sinh \lambda , {\varvec{x}}\right) \nonumber \\ \end{aligned}$$

with

$$\begin{aligned} S_{\lambda } = \{x \in {{\mathbb {R}}}^4:\,\tau (\lambda ,x)=\sinh ^2(\lambda /2)\big [(x_0-\coth (\lambda /2))^2-|{\varvec{x}}|^2\big ]=0\} \end{aligned}$$

and it is known that $O_1 \subset {{\mathbb {R}}}^4 \setminus S_\lambda$ is a $\nu _{\lambda }-$invariant set, so that the family ${\nu _{\lambda }}$, $\lambda \in {\mathbb {R}}$, acts on $O_1$ as a one-parameter group of smooth diffeomorphisms [16, Sec. 3]. Figure 1 illustrates the flow of $\nu _\lambda$, $\lambda \in {{\mathbb {R}}}$ (which is covariant under spatial rotations), on the $x_0, x_1$ plane (note that $\nu _\lambda$ extends to a diffeomorphism of the conformal compactification of ${{\mathbb {R}}}^4$ [5], but we will not need this in the following).

We now come to the announced formula for the action of the modular group. As a first step, we compute the Jacobian of the map $\nu _\lambda$.

### Lemma 2.1

For any $\lambda \in {\mathbb {R}}$ and $x\in {{\mathbb {R}}}^4{\setminus } S_\lambda$, the Jacobian determinant of $\nu _{\lambda }$ is given by2.8

### Proof

It is convenient to introduce light cone spherical coordinates $(u,v,\theta ,\varphi )$, where $\theta$, $\varphi$ are the spherical angles of ${\varvec{x}}\in {{\mathbb {R}}}^3$ and

$$\begin{aligned} u = x_0 + |{\varvec{x}}|, \quad v = x_0 - |{\varvec{x}}|. \end{aligned}$$

In these coordinates,

$$\begin{aligned} \begin{aligned} \tau (\lambda ,x)&= \frac{1}{2}(1+\cosh \lambda )-\frac{1}{2}uv(1-\cosh \lambda )-\frac{u+v}{2}\sinh \lambda \\&= \frac{1}{4e^\lambda }[(1+u)(1+v)+e^{2\lambda }(1-u)(1-v)+2e^{\lambda }(1-uv)]\\&= \frac{1}{4e^{\lambda }}[(1+u)+e^{\lambda }(1-u)]\cdot [(1+v)+e^{\lambda }(1-v)], \end{aligned} \end{aligned}$$

and the action of $\nu _\lambda$ is given by $\nu _\lambda (u,v,\theta ,\varphi ) = (u',v',\theta ',\varphi ')$, where for $\tau (\lambda , x) > 0$ one gets, from (2.7), $\theta '=\theta$, $\varphi '= \varphi$,

$$\begin{aligned} \begin{aligned} u '&= x_0'+|{\varvec{x}}'| = \frac{1}{\tau (\lambda ,x)}\left( x_0\cosh \lambda -\frac{1}{2}(1+x^2)\sinh \lambda +|{\varvec{x}}|\right) \\&= \frac{1}{2\tau (\lambda ,x)}[(u+v) \cosh \lambda -(1+uv)\sinh \lambda +u-v]\\&= \frac{1}{4e^\lambda \tau (\lambda ,x)}[(1+u)(1+v) - e^{2\lambda }(1-u)(1-v)+2e^\lambda (u-v)] \\&=\frac{(1+u)-e^\lambda (1-u)}{(1+u)+e^\lambda (1-u)}, \end{aligned} \end{aligned}$$

and, similarly,

$$\begin{aligned} v' = \frac{(1+v)-e^\lambda (1-v)}{(1+v)+e^\lambda (1-v)}, \end{aligned}$$

while for $\tau (\lambda , x) < 0$, being $|{\varvec{x}}'| = - |{\varvec{x}}|/\tau (\lambda ,x)$, one gets

$$\begin{aligned} u' = \frac{(1+v)-e^\lambda (1-v)}{(1+v)+e^\lambda (1-v)},\; v' = \frac{(1+u)-e^\lambda (1-u)}{(1+u)+e^\lambda (1-u)},\;\theta ' = \pi - \theta , \;\varphi ' = \pi +\varphi . \end{aligned}$$

By a straightforward computation the Jacobian determinant of the map $(u,v,\theta ,\varphi ) \mapsto (x_0,{\varvec{x}})$ is seen to be $\frac{1}{8}(u-v)^2 \sin \theta$. Taking then into account that

$$\begin{aligned} u'-v' = 2|{\varvec{x}}'| = \frac{2|{\varvec{x}}|}{|\tau (\lambda ,x)|} = \frac{u-v}{|\tau (\lambda ,x)|}, \end{aligned}$$

one finds

$$\begin{aligned} \begin{aligned} J_{\nu _\lambda }(x)&= \frac{(u'-v')^2}{8} \sin \theta ' \,\left( \frac{\partial u'}{\partial u} \frac{\partial v'}{\partial v}-\frac{\partial u'}{\partial v}\frac{\partial v'}{\partial u}\right) \frac{8}{(u-v)^2 \sin \theta }\\&={\textrm{sgn}}( \tau (\lambda ,x)) \frac{1}{\tau (\lambda ,x)^2}\frac{4e^\lambda }{[(1+u)+e^\lambda (1-u)]^2}\frac{4e^\lambda }{[(1+v)+e^\lambda (1-v)]^2}\\&= \frac{{\textrm{sgn}}( \tau (\lambda ,x))}{\tau (\lambda ,x)^4}. \end{aligned} \end{aligned}$$

The last equality in the statement is then a simple computation. $\square$

Observe that since $e(\lambda )$, $\lambda \in {{\mathbb {R}}}$, is a one parameter group, one getsi.e., $\nu _{-\lambda }\cdot \nu _{\lambda }=\textrm{id}$ on *T*. This entails that, for $x \in {{\mathbb {R}}}^4\setminus S_\lambda$, the limit of $\nu _{-\lambda }(\nu _\lambda (x+iy)) = x+iy$ as $y \rightarrow 0$ inside $V_+$ belongs to ${{\mathbb {R}}}^4$, and as a consequence $\nu _\lambda ({{\mathbb {R}}}^4{\setminus } S_\lambda ) \subset {{\mathbb {R}}}^4{\setminus } S_{-\lambda }$ and $\nu _{-\lambda } \cdot \nu _\lambda = \textrm{id}$ also on ${{\mathbb {R}}}^4{\setminus } S_\lambda$. It follows that $J_{\nu _{-\lambda }}(\nu _\lambda (x)) J_{\nu _\lambda }(x) = 1$ for $x \in {{\mathbb {R}}}^4\setminus S_\lambda$, so thanks to the previous lemma one deduces that $\tau (-\lambda , \nu _{\lambda }(x))$ and $\tau (\lambda ,x)$ have the same sign and

2.9 $$\begin{aligned} \tau (-\lambda , \nu _{\lambda }(x))= \frac{1}{\tau (\lambda ,x)}, \qquad x \in {{\mathbb {R}}}^4\setminus S_\lambda , \, \lambda \in {{\mathbb {R}}}. \end{aligned}$$

In view of the above, if $f \in C^\infty _c({{\mathbb {R}}}^4{\setminus } S_{2\pi \lambda },{{\mathbb {C}}}^2)$, the function $f\circ \nu _{-2\pi \lambda }$, whose support is the compact set $\nu _{2\pi \lambda }({\text {supp}\,}f) \subset {{\mathbb {R}}}^4{\setminus } S_{-2\pi \lambda }$, vanishes in a neighbourhood of $S_{-2\pi \lambda }$. Therefore for any given $\lambda \in {{\mathbb {R}}}$, the formula2.10where $c_\lambda := \cosh (\pi \lambda )$, $s_\lambda := \sinh (\pi \lambda )$, provides a well defined linear map $E_\lambda : C^\infty _c({{\mathbb {R}}}^4{\setminus } S_{2\pi \lambda },{{\mathbb {C}}}^2) \rightarrow C^\infty _c({{\mathbb {R}}}^4{\setminus } S_{-2\pi \lambda },{{\mathbb {C}}}^2)$.

From now on, we will only consider the modular operator of the algebra $\mathscr {F}(O_1)$, which we will denote simply by $\Delta$.

### Theorem 2.2

For any $\lambda \in {{\mathbb {R}}}$ and $f \in C^\infty _c({{\mathbb {R}}}^4{\setminus } S_{2\pi \lambda },{{\mathbb {C}}}^2)$, there holds

2.11 $$\begin{aligned} \Delta ^{i\lambda } \phi (f)\Omega = \phi (E_\lambda f)\Omega , \qquad \Delta ^{i\lambda } \phi (f)^*\Omega = \phi (E_\lambda f)^*\Omega , \end{aligned}$$

### Proof

We consider the vector-valued function of $y \in V_+$ defined bywhere the last equality follows from (2.5) and (2.4). Then for fixed $y' \in V_+$ we havewhich, through the change of variables $x \mapsto \nu ^{-1}_{2\pi \lambda }(x)$ becomes, thanks to Lemma 2.1,Since the integrand function has compact support in *x* independent of *y*, and is a continuous function of *x*, *y*, it can be bounded by a constant times the characteristic function of ${\text {supp}\,}(f\circ \nu _{-2\pi \lambda })$ uniformly for *y* in a neighbourhood of the origin. So, by the dominated convergence theorem, if $y \rightarrow 0$,

2.12 $$\begin{aligned} U(p(1,iy'))I_y(f) \xrightarrow [y\rightarrow 0]  \int _{{\mathbb {R}}^4} dx\, (E_\lambda f)(x)^t \phi (x+iy')\Omega , \end{aligned}$$

where (2.8) and (2.9) were used. On the other hand,

$$\begin{aligned} U(p(1,iy'))I_y(f) = U(p(1,iy'))\Delta ^{i\lambda } \int _ {{\mathbb {R}}^4} dx f(x)^t\phi (x+iy) \end{aligned}$$

that, by (2.3) and the boundedness of $U(p(1,iy'))\Delta ^{i\lambda }$, converges to $U(p(1,iy'))\Delta ^{i\lambda }\phi (f)\Omega$ as $y \rightarrow 0$. Hence, from this and (2.12)

$$\begin{aligned} U(p(1,iy'))\Delta ^{i\lambda }\phi (f)\Omega = \int _{{\mathbb {R}}^4} dx\, (E_\lambda f)(x)^t \phi (x+iy')\Omega , \end{aligned}$$

that, thanks to the strong continuity of $U(p(1,iy'))$ in $y' \in V_+$, and the fact that $E_\lambda f$ is smooth and compactly supported, implies the first equation in (2.11) in the limit $y'\rightarrow 0, y' \in V_+$. The second equation is obtained by the same argument, taking into account that for $x\in {{\mathbb {R}}}^4$ the matrixis hermitian. $\square$

### Corollary 2.3

For any $\lambda \in {{\mathbb {R}}}$ and $f \in C^\infty _c({{\mathbb {R}}}^4{\setminus } S_{2\pi \lambda },{{\mathbb {C}}}^2)$, there holds

$$\begin{aligned} \Delta ^{i\lambda }\phi (f)\Delta ^{-i \lambda }=\phi (E_\lambda f). \end{aligned}$$

### Proof

From [17, eq. (2.22)] we have

2.13 $$\begin{aligned} \phi (f)=a(L_f)+a(H_f)^* \end{aligned}$$

for specific elements  (see (3.7) below), and where $a(\cdot )$, $a(\cdot )^*$ are the usual CAR algebra annihilation and creation operators. Then, taking into account that the representation *U* of *SU*(2, 2) is of second quantization type, we have

$$\begin{aligned} \Delta ^{i\lambda }\phi (f)\Omega =[a(U(e(2\pi \lambda ))L_f)+a(U(e(2\pi \lambda ))H_f)^*]\Omega =a(U(e(2\pi \lambda ))H_f)^*\Omega , \end{aligned}$$

and on the other hand

$$\begin{aligned} \phi (E_\lambda f)\Omega =a(H_{E_\lambda f})^*\Omega , \end{aligned}$$

so that, by the previous theorem, $U(e(2\pi \lambda ))H_f=H_{E_\lambda f}$. In the same way from the action of the modular group on $\phi (f)^*\Omega$ one obtains $U(e(2\pi \lambda ))L_f=L_{E_\lambda f}$. As a consequence of these relations

$$\begin{aligned} \Delta ^{i\lambda }\phi (f)\Delta ^{-i\lambda }= a(L_{E_\lambda f})+a(H_{E_\lambda f})^*=\phi (E_\lambda f), \end{aligned}$$

and the proof is complete. $\square$

## Weyl Field Modular Group in the Cauchy Data Formulation

We denote by  the space of functions $\Phi \in C^\infty ({{\mathbb {R}}}^4,{{\mathbb {C}}}^2)$ which are solutions of the (right-handed) Weyl equation3.1with Cauchy data $\Phi _0({\varvec{x}}):= \Phi (0,{\varvec{x}})$ such that $\Phi _0 \in C^{\infty }_c({{\mathbb {R}}}^3,{{\mathbb {C}}}^2)$. The elements of  will be called (helicity 1/2, right-handed) waves, and we will frequently identify a wave with its Cauchy data. In particular, given a test function $f \in C^\infty _c({{\mathbb {R}}}^4, {{\mathbb {C}}}^2)$, the function

$$\begin{aligned} \Phi ^f(x):= \int _{{{\mathbb {R}}}^4} dy\, S(x-y) \overline{f(y)}, \qquad x \in {{\mathbb {R}}}^4 \end{aligned}$$

(convolution in the distribution sense), belongs to .

We are going to identify  with (a dense subspace of) the one particle space  of the helicity 1/2 field $\phi$ as described in [17, Sec. 2], in such a way that $\Phi ^f$ gets identified with the vector $(\phi (f)+\phi (f)^*) \Omega$.

To this end, we must first introduce some notation. For ${\varvec{p}}\in {{\mathbb {R}}}^3$ we denote by $p_\pm := (\pm |{\varvec{p}}|,{\varvec{p}})$ the corresponding 4-vectors on the future and past light cones, and the matrices  satisfy3.2showing that  are complementary orthogonal projections on ${{\mathbb {C}}}^2$, known as the projections on $\pm 1/2$ helicity spinors. Another identity which we will use in the following is3.3We will also need the spinor, defined for almost all ${\varvec{p}}\in {{\mathbb {R}}}^3$,

3.4 $$\begin{aligned} \nu _0({\varvec{p}}):= \frac{1}{2|{\varvec{p}}|\cos (\theta /2)} \left[ \begin{matrix} |{\varvec{p}}|+p_3 \\ p_1+ip_2 \end{matrix}\right] = \left[ \begin{matrix} \cos (\theta /2) \\ \sin (\theta /2) e^{i\varphi }\end{matrix}\right] , \end{aligned}$$

with $\theta \in [0,\pi ), \varphi \in [0,2\pi )$ the polar angles of ${\varvec{p}}$. One verifies that , i.e., $\nu _0({\varvec{p}})$ is a helicity 1/2 spinor. Since also $|\nu _0({\varvec{p}}) |=1$, one also obtains3.5We now denote by  the completion of the space of waves with respect to the $L^2$ norm of the Cauchy data

3.6 $$\begin{aligned} \Vert \Phi \Vert ^2 = \int _{{{\mathbb {R}}}^3} d{\varvec{x}}| \Phi _0({\varvec{x}})|^2 =\frac{1}{(2\pi )^3} \int _{{{\mathbb {R}}}^3} d{\varvec{p}}| {\hat{\Phi }}_0({\varvec{p}})|^2. \end{aligned}$$

Moreover, instead of considering the obvious complex structure on , we will consider it as a complex vector space where multiplication by the imaginary unit is defined by

$$\begin{aligned} (\imath \Phi )\hat{\phantom{a},}_0({\varvec{p}}):= \imath ({\varvec{p}}) {\hat{\Phi }}_0({\varvec{p}}), \qquad \imath ({\varvec{p}}):= \frac{i}{|{\varvec{p}}|}({\varvec{p}}\cdot {\varvec{\sigma }})=\frac{i}{|{\varvec{p}}|} \left[ \begin{matrix} p_3 &  p_1-ip_2 \\ p_1+ip_2 &  -p_3 \end{matrix}\right] . \end{aligned}$$

As a consequence, thanks to the identities becomes a complex Hilbert space with the scalar productwhich, in view of (3.2), induces the norm (3.6).

### Proposition 3.1

The map , $\Phi \mapsto (l_\Phi , h_\Phi )$, with

$$\begin{aligned} l_\Phi ({\varvec{p}}):=- \frac{1}{(2\pi )^{3/2}} \nu _0({\varvec{p}})^\dagger \hat{\Phi }_0({\varvec{p}}), \qquad h_\Phi ({\varvec{p}}):= \frac{1}{(2\pi )^{3/2}} \hat{\Phi }_0(-{\varvec{p}})^\dagger \nu _0({\varvec{p}}), \qquad {\varvec{p}}\in {{\mathbb {R}}}^3, \end{aligned}$$

is unitary and satisfies $V \Phi ^f = (\phi (f)+\phi (f)^*) \Omega$ for all $f \in C^\infty _c({{\mathbb {R}}}^4,{{\mathbb {C}}}^2)$.

### Proof

To begin with, we notice that the entries of $\nu _0({\varvec{p}})$ are bounded functions of ${\varvec{p}}$ (see Eq. (3.4)), so that *V* maps  indeed into . One then computes that, for ,where in the last equality we used (3.5). Moreover, since  under the change of variables ${\varvec{p}}\rightarrow -{\varvec{p}}$, there holdswhich inserted into the previous equation shows that $\langle V\Psi ,V\Phi \rangle = \langle \Psi , \Phi \rangle$, i.e., that *V* is isometric. Moreover,  is in the range of *V* if and only if $A({\varvec{p}}){\hat{\Phi }}_0({\varvec{p}}) = [ \psi _1({\varvec{p}}) \; \overline{\psi _2(-{\varvec{p}})}]^t$ for some , where

$$\begin{aligned} A({\varvec{p}}) = \left[ \begin{matrix} -\nu _0({\varvec{p}})^\dagger \\ \nu _0(-{\varvec{p}})^\dagger \end{matrix}\right] = \left[ \begin{matrix}- \cos (\theta /2) & -\sin (\theta /2)e^{-i\varphi }\\ \sin (\theta /2) & -\cos (\theta /2)e^{-i\varphi } \end{matrix}\right] . \end{aligned}$$

Then, since

$$\begin{aligned} A({\varvec{p}})^{-1} = \left[ \begin{matrix} -\cos (\theta /2) & \sin (\theta /2)\\ -\sin (\theta /2) e^{i\varphi }& -\cos (\theta /2)e^{i\varphi } \end{matrix}\right] \end{aligned}$$

has bounded entries, we conclude that *V* maps  onto  and is then unitary.

In order to prove the last statement, we first observe that for $f \in C^\infty _c({{\mathbb {R}}}^4, {{\mathbb {C}}}^2)$,

$$\begin{aligned} (\phi (f)+\phi (f)^*) \Omega = (l_f, h_f) \in L^2({{\mathbb {R}}}^3) \oplus L^2({{\mathbb {R}}}^3), \end{aligned}$$

with

3.7 $$\begin{aligned} l_f({\varvec{p}}):= -\frac{1}{(2\pi )^{3/2}} \hat{\bar{f}}(p_+)_{\alpha }\overline{\nu _0({\varvec{p}})_{\alpha }}, \qquad h_f({\varvec{p}}):= \frac{1}{(2\pi )^{3/2}} \hat{f}(p_+)_{\alpha }\nu _0({\varvec{p}})_{\alpha }. \end{aligned}$$

Then, we computewhich, in view of (3.2), entails3.8From the first one of the above equations, taking into account the hermiticity of  and the fact, observed above, that $\nu _0({\varvec{p}})$ has helicity 1/2, we then getwhich entails $l_f = l_{\Phi ^f}$. Similarly, making the substitution ${\varvec{p}}\rightarrow -{\varvec{p}}$ in the second equation in (3.8) one obtains $\hat{f}(p_+)_{\alpha }\nu _0({\varvec{p}})_{\alpha } = \hat{\bar{\Phi }}^f_0({\varvec{p}})_\alpha \nu _0({\varvec{p}})_{\alpha }$ and therefore $h_f = h_{\Phi ^f}$. $\square$

In view of the above discussion, we can now compute the action of the modular group on waves by pulling back its action on  obtained in Thm. 2.2. In the following, we will directly identify  with , avoiding to mention explicitly the unitary *V*, whenever this does not cause confusion.

### Theorem 3.2

Given  there is $\varepsilon > 0$ such that for $|\lambda | < \varepsilon$,  and3.9

### Proof

For  there is an open ball $B_r \subset {{\mathbb {R}}}^3$ of radius $r > 0$ centered at the origin which contains the support of $\Phi _0$. Then, arguing as in the proof of [12, Lemma A.3], we can find $f \in C^\infty _c(O_r,{{\mathbb {C}}}^2)$, $O_r \subset {{\mathbb {R}}}^4$ the double cone with basis $B_r$, such that $\Phi = \Phi ^f$. Now for sufficiently small $|\lambda |$ one has $B_r \subset {{\mathbb {R}}}^4 {\setminus } S_{2\pi \lambda }$, and as a consequence, by the above discussion and Thm. 2.2, $\Delta ^{i\lambda } \Phi = \Phi ^{E_\lambda f}$, which in particular entails that  is smooth on ${{\mathbb {R}}}^4$. Moreover if $g \in C^\infty _c({{\mathbb {R}}}^4{\setminus } S_{-2\pi \lambda }, {{\mathbb {C}}}^2)$, one has, thanks to (2.1), Cor. 2.3 and (2.10),Using then the change of variable $x \rightarrow \nu _{-2\pi \lambda }(x)$, Lemma 2.1 and Eq. (2.9), we getand formula (3.9) follows by the arbitrariness of *g*. $\square$

We can now compute the action of the modular hamiltonian $\log \Delta = -i \frac{d}{d\lambda } \Delta ^{i\lambda } |_{\lambda = 0}$ on waves . To begin with, we notice that from (2.6), (2.7) it follows

$$\begin{aligned} \left. \frac{d}{d\lambda } \tau (-2\pi \lambda ,x)\right| _{\lambda = 0}&= 2\pi x_0, \quad x \in {{\mathbb {R}}}^4,\\ \left. \frac{d}{d\lambda } \nu _{-2\pi \lambda }(x)\right| _{\lambda = 0}&=\pi (1-x_0^2-|{\varvec{x}}|^2,- 2x_0 {\varvec{x}}), \quad x \in {{\mathbb {R}}}^4\setminus S_{-2\pi \lambda }. \end{aligned}$$

### Lemma 3.3

For any $x\in {{\mathbb {R}}}^4$, we have:

### Proof

By the above formulas, there holds$\square$

### Theorem 3.4

The one particle modular hamiltonian of the unit double cone for the helicity 1/2 field on the wave space  is given by $\log \Delta = - \iota K$, where for each

3.10 $$\begin{aligned} \begin{aligned}&(K\Phi )_0({\varvec{x}}) = -\pi \big [(1-r^2) {\varvec{\sigma }}\cdot \nabla \Phi _0({\varvec{x}}) - {\varvec{x}}\cdot {\varvec{\sigma }}\Phi _0({\varvec{x}}) \big ]\\&\quad = -\pi \left[ \begin{matrix}(1-r^2)\partial _3-x_3 &  (1-r^2)(\partial _1-i\partial _2)-x_1+ix_2 \\ (1-r^2)(\partial _1+i\partial _2)-x_1-ix_2 & -(1-r^2)\partial _3+x_3 \end{matrix}\right] \Phi _0({\varvec{x}}), \end{aligned}\nonumber \\ \end{aligned}$$

with $$r=|{\varvec{x}}|$$.

### Proof

Given , we need to check that

3.11 $$\begin{aligned}  &   \lim _{\lambda \rightarrow 0}\left\| K\Phi -\frac{1}{\lambda }(\Delta ^{i\lambda }\Phi -\Phi ) \right\| ^2 \nonumber \\  &   \quad =\lim _{\lambda \rightarrow 0}\int _{{{\mathbb {R}}}^3} d{\varvec{x}}\, \left| (K\Phi )_0({\varvec{x}})-\frac{1}{\lambda }\left[ (\Delta ^{i\lambda }\Phi )_0({\varvec{x}}) -\Phi _0({\varvec{x}})\right] \right| ^2 = 0. \end{aligned}$$

To this end, using the previous formulas and (3.9), we compute, for fixed $x \in {{\mathbb {R}}}^4 \setminus S_{-2\pi \lambda }$,where in the last equality we used the Weyl equation (3.1). Thus for $|{\varvec{x}}| \ne |\coth (\pi \lambda )|$,

$$\begin{aligned} \left. \frac{d}{d\lambda } (\Delta ^{i\lambda }\Phi )_0({\varvec{x}}) \right| _{\lambda = 0} = -\pi \big [(1-r^2) {\varvec{\sigma }}\cdot \nabla \Phi _0({\varvec{x}}) - {\varvec{x}}\cdot {\varvec{\sigma }}\Phi _0({\varvec{x}}) \big ], \end{aligned}$$

and as a consequence, to conclude the proof it is sufficient to show that the support of the integrand in (3.11) is contained, for sufficiently small $|\lambda |$, in the ball of radius $|\coth (\pi \lambda )|$ centered at the origin, and that it is possible to interchange the limit and the integral. We show in Appendix A that this can actually be done by an application of the dominated convergence theorem. $\square$

## Modular Group for Massless Dirac and Majorana Fields

If $\phi$ is a right-handed helicity 1/2 field, it is immediate to verify that $\chi (x_0, {\varvec{x}}):= \phi (x_0, -{\varvec{x}})$ is a left-handed helicity 1/2 field, i.e., it satisfies the left-handed Weyl equation

$$\begin{aligned} {\tilde{\partial }} \chi (x) = (\partial _0 - \sigma _j \partial _j)\chi (x) = 0, \end{aligned}$$

and the anticommutation relationsIt is then immediate, using the results of the previous sections, to obtain the action of the modular group on $\chi$ and on the corresponding waves’ space. We therefore avoid to write down explicit formulas.

If now $\phi$ and $\chi$ are independent right-handed and left-handed helicity 1/2 fields respectively, with respective one particle spaces  and  (both copies of $L^2({{\mathbb {R}}}^3) \oplus L^2({{\mathbb {R}}}^3)$), it is easy to check that the 4-component field

4.1 $$\begin{aligned} \psi (x):= \left[ \begin{matrix} \phi (x) \\ \chi (x)\end{matrix}\right] , \end{aligned}$$

acting on the fermionic Fock space over , satisfies the massless Dirac equation

$$\begin{aligned} i \gamma ^\mu \partial _\mu \psi (x) = 0 \end{aligned}$$

with the (chiral) gamma matrices

4.2 $$\begin{aligned} \gamma ^0 = \left[ \begin{matrix} 0 & -1 \\ -1 & 0\end{matrix}\right] , \qquad \gamma ^k = \left[ \begin{matrix} 0 & \sigma _k \\ -\sigma _k & 0\end{matrix}\right] , \quad k=1,2,3, \end{aligned}$$

and satisfies the anticommutation relations

$$\begin{aligned} \{ \psi _\alpha (x),\psi ^*_\beta (y)\}=(\gamma ^\mu \gamma ^0)_{\alpha \beta }\partial _\mu D(x-y), \qquad \alpha ,\beta =1,\ldots ,4. \end{aligned}$$

The Dirac field then defines the twisted local net of von Neumann algebrasIt is then easy to obtain the description of the one particle space of the field $\psi$ in terms of classical solutions of the massless Dirac equation (Dirac waves), and the action on Dirac waves of the modular hamiltonian of the unit double cone, from the corresponding statements for the components Weyl fields $\phi$ and $\chi$, obtained above. We will therefore limit ourselves to state the main results, refraining from giving the straightforward proofs.

Let then  denote the space of $C^\infty ({{\mathbb {R}}}^4,{{\mathbb {C}}}^4)$ solutions $\Psi$ of the massless Dirac equation with Cauchy data $\Psi _0:= \Psi (0,\cdot ) \in C^\infty _c({{\mathbb {R}}}^3,{{\mathbb {C}}}^4)$. Its completion  with respect to the Cauchy data $L^2$ norm is a complex Hilbert space with the scalar productwhere for $p \in {{\mathbb {R}}}^4$, , and multiplication by the imaginary unit defined by

$$\begin{aligned} (\imath \Psi )\hat{\phantom{a},}_0({\varvec{p}}):= i\frac{p_k}{|{\varvec{p}}|}\gamma ^0\gamma ^k {\hat{\Psi }}_0({\varvec{p}}). \end{aligned}$$

Using Prop. 3.1, and its counterpart for the left handed Weyl fied, the space of Dirac waves  is then naturally identified with the one particle space .

As a consequence of Thm. 3.4 (and of its left-handed version), the modular hamiltonian then acts on Dirac waves as follows.

### Theorem 4.1

The one particle modular hamiltonian of the unit double cone for the massless Dirac field on the Dirac wave space  is given by $\log \Delta = - \imath K_D$, where for each

4.3 $$\begin{aligned} (K_D\Psi )_0({\varvec{x}}) = -\pi \big [(1-r^2) \partial _k - x_k\big ] \gamma ^0\gamma ^k \Psi _0({\varvec{x}}). \end{aligned}$$

It is also worthwhile to consider a massless Majorana field $\psi$, i.e., a massless Dirac field satisfying the reality condition

$$\begin{aligned} \gamma ^0 C \psi ^*(x) = \psi (x), \qquad C:= \left[ \begin{matrix}- \sigma _2 & 0 \\ 0 & \sigma _2\end{matrix}\right] . \end{aligned}$$

As such, it can be obtained as the particular case of (4.1) where  and $\chi = \sigma _2 \phi ^*$. This implies the identity

4.4 $$\begin{aligned} \psi (f)+\psi (f)^*= \phi (g + \sigma _2 {\bar{h}}) + \phi (g+\sigma _2 {\bar{h}})^*, \quad f= \left[ \begin{matrix} g \\ h\end{matrix}\right] , \; g, h \in C^\infty _c({{\mathbb {R}}}^4,{{\mathbb {C}}}^2), \end{aligned}$$

showing that the local von Neumann algebras, and then the modular groups, of the massless Majorana and of the helicity 1/2 fields coincide; i.e., the massless Majorana field is just a 4-component reformulation of the Weyl field.

The corresponding one particle Hilbert space  can of course also be written in the 4-component formalism aswhich, thanks to the identity $C \gamma ^0 {\bar{\gamma }}^k = \gamma ^k \gamma ^0 C$, is a complex closed subspace of  (i.e.,  implies ), and it is also invariant under the operator $K_D$. Indeed, one easily verifies, using the identity (3.3), that the unitary map identifying  with  is4.5and it is immediate to see that it intertwines the action of *K* in (3.10) with the restriction of that of $K_D$ in (4.3) to . The latter provides then the modular hamiltonian of the unit double cone for the massless Majorana field.

As an application of our formula for the local modular hamiltonian of the Majorana field, the relative entropy of suitable one particle states with respect to the vacuum on the local algebra $\mathscr {F}(O_1)$ can be computed. To this end, if , we denote by $\omega _\Psi = \Vert \Psi \Vert ^{-2}\langle \Psi , (\cdot )\Psi \rangle$ the corresponding vector state on the algebra of bounded operators on the antisymmetric Fock space on . In particular $\omega = \langle \Omega , (\cdot )\Omega \rangle$ is the vacuum state. We also denote by  the set of smooth elements .

### Proposition 4.2

Let  be such that $\operatorname {supp} \Psi _0 \subset B$ and $\Vert \Psi \Vert = 1$. The relative entropy of $\omega _\Psi$ with respect to $\omega$ on $\mathscr {F}(O_1)$ is given by

$$\begin{aligned} S(\omega _\Psi \Vert \omega )= &   \frac{1}{4\pi ^2} \int \frac{d{\varvec{p}}}{|{\varvec{p}}|} \big [{\hat{\Psi }}_0({\varvec{p}})^\dagger |{\varvec{p}}|^2(1+\nabla ^2) {\hat{\Psi }}_0({\varvec{p}}) \\  &   + {\hat{\Psi }}_0({\varvec{p}})^\dagger {\varvec{p}}\cdot \nabla {\hat{\Psi }}_0({\varvec{p}})+ i p_j {\hat{\Psi }}_0({\varvec{p}})^\dagger \sigma ^{jk} \partial _k {\hat{\Psi }}_0({\varvec{p}})\big ], \end{aligned}$$

where $$\sigma ^{jk} = \frac{i}{2}[\gamma ^j,\gamma ^k]$$.

### Proof

Analogously to the proof of Thm. 3.2, from $\operatorname {supp} \Psi _0 \subset B$ it follows that we can find $f \in C_c^\infty (O_1,{{\mathbb {C}}}^4)$ such that

$$\begin{aligned} \Psi (x) = \Psi ^f(x):= \int _{{{\mathbb {R}}}^4} dy \,\gamma ^\mu \gamma ^0 \partial _\mu D(x-y) \overline{f(y)}. \end{aligned}$$

Moreover, one computes easily that $\gamma ^0 C \overline{\Psi ^f} = \Psi ^{-\gamma ^0 C {\bar{f}}}$, so that, since , we can also choose *f* such that $\gamma ^0 C {\bar{f}} = -f$. This in turn entails

$$\begin{aligned} f = \left[ \begin{matrix} g \\ -\sigma _2 {\bar{g}}\end{matrix}\right] \quad \text {and}\quad \Psi ^f = \left[ \begin{matrix} \Phi ^g \\ \sigma _2 \overline{\Phi ^g}\end{matrix}\right] \end{aligned}$$

with $g \in C_c^\infty (O_1,{{\mathbb {C}}}^2)$, so that, thanks to (4.4), to Prop. 3.1 and to the identification (4.5),

$$\begin{aligned} (\psi (f)+\psi (f)^*)\Omega = 2(\phi (g)+\phi (g)^*)\Omega \cong 2\Phi ^g \cong \sqrt{2}\Psi ^f. \end{aligned}$$

Moreover, from Eq. (2.13) and the CARs, one obtains

$$\begin{aligned} \begin{aligned} \{\psi (f)+\psi (f)^*, \psi (f)+\psi (f^*)\}&= 4\{\phi (g)+\phi (g)^*,\phi (g)+\phi (g)^*\} \\&= 8 \{a(l_g,h_g)^*,a(l_g,h_g)\}\\&= 8\Vert (l_g,h_g)\Vert ^2 = 8 \Vert \Phi ^g\Vert ^2 = 4 \Vert \Psi ^f\Vert ^2 = 4, \end{aligned} \end{aligned}$$

i.e., the operator $B(f):=2^{-1/2} (\psi (f)+\psi (f)^*) \in \mathscr {F}(O_1)$ is unitary. As a consequence, the unit vector $\Psi = B(f)\Omega$ is cyclic and separating for $\mathscr {F}(O_1)$, and for the one parameter group generated by the relative modular operator $\Delta _{\Psi , \Omega }$ one has [7, Lemma 5.7]

$$\begin{aligned} \Delta _{\Psi , \Omega }^{i\lambda } = B(f) \Delta ^{i\lambda } B(f)^*, \qquad \lambda \in {{\mathbb {R}}}. \end{aligned}$$

Therefore the relative entropy between $\omega _\Psi$ and $\omega$ on $\mathscr {F}(O_1)$ is given by

$$\begin{aligned} \begin{aligned} S(\omega _\Psi \Vert \omega )&= -\langle \Omega , \log \Delta _{\Psi ,\Omega }\Omega \rangle = i \frac{d}{d\lambda } \langle \Omega , B(f) \Delta ^{i\lambda }B(f)^*\Omega \rangle \Big |_{\lambda = 0} \\&= 2i \langle \Psi , K_D\Psi \rangle = -2 {\textrm{Im}\,}\langle \Psi , K_D\Psi \rangle , \end{aligned} \end{aligned}$$

where the last equality is due to the skew-selfadjointness of $K_D$. The statement is then obtained by Lemma B.1. $\square$

The formula for the relative entropy just obtained can be recast in a more familiar form by recalling that the quantum energy density of the Majorana field is expressed by

4.6 $$\begin{aligned} T_{00}(x)&= \frac{i}{2}: \psi ^\dagger (x) \partial _0 \psi (x) - \partial _0 \psi ^\dagger (x) \psi (x) \nonumber : \\  &= \frac{i}{2}:\partial _k \psi ^\dagger (x) \gamma ^0 \gamma ^k \psi (x) - \psi ^\dagger (x) \gamma ^0 \gamma ^k \partial _k \psi (x): \end{aligned}$$

as a quadratic form on a domain which can be taken to be the linear span in  of  (antisymmetric tensor powers).

### Theorem 4.3

Let  be such that $\operatorname {supp} \Psi _0 \subset B$ and $\Vert \Psi \Vert =1$. Then

$$\begin{aligned} S(\omega _\Psi \Vert \omega ) =\frac{1}{4\pi ^2} \int _{{{\mathbb {R}}}^3} d{\varvec{x}}\,(1-r^2) \langle \Psi , T_{00}(0,{\varvec{x}}) \Psi \rangle . \end{aligned}$$

### Proof

Using the notation $t({\varvec{x}}):= \langle \Psi , T_{00}(0,{\varvec{x}}) \Psi \rangle$ one gets, by Lemma B.2,

$$\begin{aligned} \begin{aligned} {\hat{t}}({\varvec{p}})&= \frac{1}{2(2\pi )^3} \int d{\varvec{q}}\bigg \{\big (|{\varvec{q}}|+|{\varvec{q}}+{\varvec{p}}|\big ) {\hat{\Psi }}_0({\varvec{q}})^\dagger {\hat{\Psi }}_0({\varvec{q}}+{\varvec{p}})\\&\quad + \left( \frac{1}{|{\varvec{q}}|}+\frac{1}{|{\varvec{q}}+{\varvec{p}}|}\right) {\hat{\Psi }}_0({\varvec{q}})^\dagger \big [{\varvec{q}}\cdot ({\varvec{q}}+{\varvec{p}})+i ({\varvec{q}}\wedge {\varvec{p}})\cdot \varvec{\Sigma }\big ]{\hat{\Psi }}_0({\varvec{q}}+{\varvec{p}})\bigg \}, \end{aligned} \end{aligned}$$

and therefore, by elementary computations and from Prop. 4.2,

$$\begin{aligned} \int _{{{\mathbb {R}}}^3} d{\varvec{x}}\, (1-r^2) t({\varvec{x}}) =(2\pi )^3 (1+ \nabla ^2) {\hat{t}}(\varvec{0}) = 4\pi ^2S(\omega _\Psi \Vert \omega ), \end{aligned}$$

which proves the statement. $\square$

The above result is coherent with the fact that $\log \Delta = \frac{1}{2}( H-C_0)$, with *H* the Hamiltonian and $C_0$ the time component of the generator of special conformal trasformations [17, Thm. 4.8], which is expected to be related to the energy density by

$$\begin{aligned} C_0 = \int _{{{\mathbb {R}}}^3} d{\varvec{x}}\, r^2 T_{00}(0,{\varvec{x}}) \end{aligned}$$

(see, e.g., [14, Eq. (2.77)]), by the classical Noether theorem and the tracelessness of the massless Majorana field energy-momentum tensor. However, to the best of our knowledge, no proof of this formula in quantum field theory is available in the literature.

We can also obtain the relative entropy between the restrictions of $\omega _\Psi$ and $\omega$ to the observable von Neumann algebra $\mathscr {A}(O_1)$ of the massless Majorana field. This is defined as the fixed point subalgebra of $\mathscr {F}(O_1)$ with respect to the involutive automorphism $\beta : \mathscr {F}(O_1) \rightarrow \mathscr {F}(O_1)$ such that

$$\begin{aligned} \beta (\psi (f)) = -\psi (f), \qquad f \in C^\infty _c({{\mathbb {R}}}^4,{{\mathbb {C}}}^4), \end{aligned}$$

i.e., $$\beta = \operatorname {Ad} U(p(-1,0))$$.

### Corollary 4.4

Let  be such that $\operatorname {supp} \Psi _0 \subset B$ and $\Vert \Psi \Vert =\frac{1}{ \sqrt{2}}$. Then

$$\begin{aligned} S\big (\omega _\Psi |_{\mathscr {A}(O_1)}\,\big \Vert \,\omega |_{\mathscr {A}(O_1)}\big ) = S(\omega _\Psi \Vert \omega ). \end{aligned}$$

### Proof

We denote by $E: \mathscr {F}(O_1) \rightarrow \mathscr {A}(O_1)$ the normal conditional expectation

$$\begin{aligned} E(\psi ):= \frac{1}{2}(\psi + \beta (\psi )), \qquad \psi \in \mathscr {F}(O_1). \end{aligned}$$

Since $\omega \circ E = \omega$ and $\omega$ is a faithful state on $\mathscr {F}(O_1)$, *E* is faithful too. Moreover from

$$\begin{aligned} U(p(-1,0))\Psi = U(p(-1,0))B(f)\Omega = - B(f)\Omega = -\Psi \end{aligned}$$

one gets $\omega _\Psi (\beta (\psi )) = \omega _\Psi (\psi )$ for all $\psi \in \mathscr {F}(O_1)$, i.e., $\omega _\Psi \circ E = \omega _\Psi$. The statement then follows from [20, Thm. 5.15]. $\square$

An obvious extension of the above results would be to compute the relative entropy between the vacuum and a one particle state not localized in *B*. While it is fairly easy to verify that if such a state is localized in the complement of *B* the relative entropy vanishes (e.g., since the relative modular operator coincides with $\Delta$), already for states which are sums of a state localized in *B* and one in its complement the problem becomes much more difficult. We plan to come back to these issues in the future.

## Data Availability

Data sharing is not applicable to this article as no datasets were generated or analysed during the current study.

## Estimates for Thm. 3.4

We show that it is possible to apply the dominated convergence theorem to interchange the limit and integral in (3.11) in the proof of Thm. 3.4.

To this end, let $R > 0$ be such that ${\text {supp}\,}\Phi _0 \subset B_R$. It is then easy to see that there is an $\varepsilon > 0$ such that for $|\lambda | < \varepsilon$ there holds ${\text {supp}\,}(\Delta ^{i\lambda }\Phi )_0 \subset B_{2R}$. Indeed, in view of (3.9), ${\text {supp}\,}(\Delta ^{i\lambda }\Phi )_0 = \{ {\varvec{x}}\in {{\mathbb {R}}}^3\,:\, \nu _{-2\pi \lambda }(0,{\varvec{x}}) \in {\text {supp}\,}\Phi \}$ and one has, thanks to Huyghens’ principle, that ${\text {supp}\,}\Phi$ is defined, in the light cone spherical coordinates introduced in the proof of Lemma 2.1, by the inequalities $v \le u$ and $-R \le v \le R$, or $-R \le u \le R$. On the other hand, still in these coordinates, one checks that on the time zero plane

$$\begin{aligned} \tau (-2\pi \lambda ,(u,-u,\theta ,\varphi )) > 0 \quad \Leftrightarrow \quad 0 \le u < |\coth (\pi \lambda )|, \end{aligned}$$

so that $\nu _{-2\pi \lambda }$ acts on such plane as

$$\begin{aligned}  &   \nu _{-2\pi \lambda }(u,-u,\theta ,\varphi ) \\    &   \quad = {\left\{ \begin{array}{ll} (f_{-2\pi \lambda }(u), f_{-2\pi \lambda }(-u), \theta , \varphi ), & \text {if }0 \le u < |\coth (\pi \lambda )|,\\ (f_{-2\pi \lambda }(-u), f_{-2\pi \lambda }(u), \pi - \theta , \pi + \varphi ), & \text {if }u > |\coth (\pi \lambda )|, \end{array}\right. } \end{aligned}$$

with

$$\begin{aligned} f_{\lambda }(u):= \frac{(1+u)-e^\lambda (1-u)}{(1+u)+e^\lambda (1-u)}. \end{aligned}$$

If then $\varepsilon$ is such that $\coth (\pi \varepsilon ) > R$, and such that for $|\lambda | < \varepsilon$, there holds $|\coth (\pi \lambda )| > \max \{ f_{2\pi \lambda }(R), -f_{2\pi \lambda }(-R)\}$ and $f_{2\pi \lambda }(R) < 2R$ (which is possible since $f_{2\pi \lambda }(R) \rightarrow R$ as $\lambda \rightarrow 0$), one sees, by the monotonicity properties of $f_{-2\pi \lambda } = f_{2\pi \lambda }^{-1}$, that for $|\lambda | < \varepsilon$

$$\begin{aligned}&f_{2\pi \lambda }(R)< u< |\coth (\pi \lambda )| \quad \Rightarrow \quad f_{-2\pi \lambda }(u)> R,\, f_{-2\pi \lambda }(-u)< -R,\\&u> |\coth (\pi \lambda )| \quad \Rightarrow \quad {\left\{ \begin{array}{ll} f_{-2\pi \lambda }(u)> f_{-2\pi \lambda }(\coth (\pi \lambda ))> R & \text {if }\lambda > 0,\\ f_{-2\pi \lambda }(-u)< f_{-2\pi \lambda }(\coth (\pi \lambda ))< -R & \text {if }\lambda < 0. \end{array}\right. } \end{aligned}$$

As a consequence ${\text {supp}\,}(\Delta ^{i\lambda }\Phi )_0$ is contained in the subset of the time zero plane defined by $0 \le u \le f_{2\pi \lambda }(R)$, which is in turn contained in $B_{2R}$.

Since clearly ${\text {supp}\,}(K\Phi )_0 \subset B_R$, it is then sufficient to show that for all ${\varvec{x}}\in B_{2R}$ and for $|\lambda | < \varepsilon$,

$$\begin{aligned} \left| (K\Phi )_0({\varvec{x}})-\frac{1}{\lambda }[(\Delta ^{i\lambda }\Phi )_0({\varvec{x}}) -\Phi _0({\varvec{x}})] \right| \le C, \end{aligned}$$

where $C > 0$ is a constant independent from $\lambda$. In order to do this, we first observe that we can choose $\varepsilon > 0$ such that for $|\lambda | < \varepsilon$ there also holds $f_{4\pi \lambda }(R) < 2R$. This implies that if $|\lambda | < \varepsilon$, for all ${\varvec{x}}\in {\text {supp}\,}(\Delta ^{i\lambda }\Phi )_0$ one has $\nu _{-2\pi \lambda }(0,{\varvec{x}}) \in O_{2R}$, the double cone with basis $B_{2R}$. Indeed, if $0 \le u \le f_{2\pi \lambda }(R) < |\coth (\pi \lambda )|$, then

$$\begin{aligned} f_{-2\pi \lambda }(u) \le R < 2R, \quad f_{-2\pi \lambda }(-u) \ge f_{-2\pi \lambda }(-f_{2\pi \lambda }(R)) = -f_{4\pi \lambda }(R) > -2R, \end{aligned}$$

and in light cone spherical coordinates $O_{2R}$ is given by $- 2R< v \le u < 2R$.

Then, from (3.10) and (3.9), and using the last observation, we get the estimate

$$\begin{aligned} \begin{aligned}&\bigg | (K\Phi )_0({\varvec{x}})-\frac{1}{\lambda }\left[ (\Delta ^{i\lambda }\Phi )_0({\varvec{x}}) -\Phi _0({\varvec{x}})\right] \bigg | \\&\quad =\bigg |\pi {\varvec{x}}\cdot {\varvec{\sigma }}\big [\Phi _0({\varvec{x}})-\Phi (\nu _{-2 \pi \lambda }(0,{\varvec{x}}))\big ]\\&\qquad -\frac{1}{\lambda }\left[ \Phi (\nu _{-2 \pi \lambda }(0,{\varvec{x}}))-\Phi _0({\varvec{x}})\right] +\pi (1-r^2)\partial _0 \Phi (0,{\varvec{x}})\\&\qquad +\left[ \pi {\varvec{x}}\cdot {\varvec{\sigma }}-\frac{1}{\lambda }\left( \frac{c_\lambda +s_\lambda {\varvec{x}}\cdot {\varvec{\sigma }}}{c_\lambda ^2-r^2s_\lambda ^2}-1\right) \right] \Phi (\nu _{-2\pi \lambda }(0,{\varvec{x}}))\bigg | \\&\quad \le \left[ 4\pi R \sum _{j=1}^3 \Vert \sigma _j\Vert +\bigg \Vert \pi {\varvec{x}}\cdot {\varvec{\sigma }}-\frac{1}{\lambda }\left( \frac{c_\lambda +s_\lambda {\varvec{x}}\cdot {\varvec{\sigma }}}{c_\lambda ^2-r^2s_\lambda ^2}-1\right) \bigg \Vert \right] \sup _{O_{2R}} |\Phi | \\&\qquad + \bigg |\frac{1}{\lambda }\left[ \Phi (\nu _{-2 \pi \lambda }(0,{\varvec{x}}))-\Phi (0,{\varvec{x}})\right] -\pi (1-r^2)\partial _0 \Phi (0,{\varvec{x}})\bigg |, \end{aligned} \end{aligned}$$

where as usual $r = |{\varvec{x}}|$. Next, we can further estimate

$$\begin{aligned} \begin{aligned}&\bigg \Vert \pi {\varvec{x}}\cdot {\varvec{\sigma }}-\frac{1}{\lambda }\left( \frac{c_\lambda +s_\lambda {\varvec{x}}\cdot {\varvec{\sigma }}}{c_\lambda ^2-r^2s_\lambda ^2}-1\right) \bigg \Vert \\&\quad = \left\| \frac{1}{\lambda }(c_\lambda -1) +\frac{1}{\lambda }(s_\lambda -\pi \lambda ){\varvec{x}}\cdot {\varvec{\sigma }}+\frac{1}{\lambda }(c_\lambda +s_\lambda {\varvec{x}}\cdot {\varvec{\sigma }})\left( \frac{1}{c_\lambda ^2-r^2s_\lambda ^2}-1\right) \right\| \\&\quad \le \left| \frac{1}{\lambda }(c_\lambda -1)\right| +\left| \frac{1}{\lambda }(s_\lambda -\pi \lambda )\right| \Vert {\varvec{x}}\cdot {\varvec{\sigma }}\Vert \\&\qquad +(|c_\lambda |+|s_\lambda | \Vert {\varvec{x}}\cdot {\varvec{\sigma }}\Vert )\frac{1}{|c_\lambda ^2-r^2 s^2_\lambda |}\left( \left| \frac{1}{\lambda }(c_\lambda ^2-1)\right| +r^2 \frac{s_\lambda ^2}{\lambda }\right) , \end{aligned} \end{aligned}$$

and since we can choose $\varepsilon$ such that if $|\lambda | < \varepsilon$ one has $\frac{2c_\lambda ^2-1}{2s_\lambda ^2} > 4R^2$, which entails $c_\lambda ^2-r^2s_\lambda ^2 \ge \frac{1}{2}$ for $r < 2R$, we see that the last line can be bounded by a constant uniformly for $|\lambda | < \varepsilon$ and $r < 2R$.

For the remaining term, by Lagrange’s theorem we can find a $\xi _\lambda$ between $(0,{\varvec{x}})$ and $\nu _{-2\pi \lambda }(0,{\varvec{x}})$ such that, using also (2.7),

$$\begin{aligned} \begin{aligned}&\bigg |\frac{1}{\lambda }\big [\Phi _\alpha (\nu _{-2 \pi \lambda }(0,{\varvec{x}}))-\Phi _\alpha (0,{\varvec{x}})\big ]-\pi (1-r^2)\partial _0 \Phi _\alpha (0,{\varvec{x}})\bigg |=\\&\quad = \bigg |(1-r^2)\partial _0\Phi _\alpha (\xi _\lambda )\left[ \frac{s_\lambda }{\lambda }\frac{c_\lambda }{c_\lambda ^2-r^2s_\lambda ^2}-\pi \right] +x_k\partial _k\Phi _\alpha (\xi _\lambda )\frac{1}{\lambda }\left( \frac{1}{c_\lambda ^2-r^2s_\lambda ^2}-1\right) \\&\qquad +\pi (1-r^2)\left[ \partial _0\Phi _\alpha (\xi _\lambda )-\partial _0 \Phi _\alpha (0,{\varvec{x}})\right] \bigg |\\&\quad \le (4R^2-1)\sup _{O_{2R}} |\partial _0\Phi _\alpha |\left( \left| \frac{s_\lambda }{\lambda }\frac{c_\lambda }{c_\lambda ^2-r^2s_\lambda ^2}-\pi \right| +2\pi \right) \\&\qquad +2R \sup _{k,O_{2R}} |\partial _k\Phi _\alpha | \left| \frac{1}{\lambda }\left( \frac{1}{c_\lambda ^2-r^2s_\lambda ^2}-1\right) \right| , \end{aligned} \end{aligned}$$

and, arguing as above, it is then easy to see that also this term can be estimated by a fixed constant uniformly for $|\lambda | < \varepsilon$ and $r < 2R$, thus concluding the proof.

## Computations for the Relative Entropy of the Majorana Field

### Lemma B.1

For , there holds

$$\begin{aligned} {\textrm{Im}\,}\langle \Psi , K_D\Psi \rangle= &   - \frac{1}{8\pi ^2} \int \frac{d{\varvec{p}}}{|{\varvec{p}}|} \big [{\hat{\Psi }}_0({\varvec{p}})^\dagger |{\varvec{p}}|^2(1+\nabla ^2) {\hat{\Psi }}_0({\varvec{p}}) \\  &   + {\hat{\Psi }}_0({\varvec{p}})^\dagger {\varvec{p}}\cdot \nabla {\hat{\Psi }}_0({\varvec{p}})+ i p_j {\hat{\Psi }}_0({\varvec{p}})^\dagger \sigma ^{jk} \partial _k {\hat{\Psi }}_0({\varvec{p}})\big ]. \end{aligned}$$

### Proof

From the identity  one gets

B.1 $$\begin{aligned} \begin{aligned} {\textrm{Im}\,}\langle \Psi , K_D\Psi \rangle&= \frac{1}{2i} \big ( \langle \Psi , K_D\Psi \rangle - \langle K_D\Psi , \Psi \rangle \big ) \\&= \frac{1}{2i(2\pi )^3} \int _{{{\mathbb {R}}}^3} \frac{d{\varvec{p}}}{|{\varvec{p}}|}p_k\big [ {\hat{\Psi }}_0({\varvec{p}})^\dagger \gamma ^0 \gamma ^k \widehat{(K_D\Psi )}_0({\varvec{p}})\\&- \widehat{(K_D\Psi )}_0({\varvec{p}})^\dagger \gamma ^0 \gamma ^k {\hat{\Psi }}_0({\varvec{p}})\big ]. \end{aligned} \end{aligned}$$

Moreover from Thm. 4.1 and using $\nabla ^2( p_j {\hat{\Psi }}_0) = 2 \partial _j{\hat{\Psi }}_0 + p_j \nabla ^2 {\hat{\Psi }}_0$ (where all the derivatives are with respect to the components of ${\varvec{p}}$),

$$\begin{aligned} \begin{aligned} \widehat{(K_D\Psi )}_0({\varvec{p}})&= -\pi \gamma ^0 \gamma ^j \left[ (1+\nabla ^2)(ip_j {\hat{\Psi }}_0({\varvec{p}}))-i\partial _j {\hat{\Psi }}_0({\varvec{p}})\right] \\&= -i \pi \gamma ^0 \gamma ^j \left( p_j \nabla ^2 {\hat{\Psi }}_0({\varvec{p}}) + \partial _j {\hat{\Psi }}_0({\varvec{p}})+p_j {\hat{\Psi }}_0({\varvec{p}})\right) . \end{aligned} \end{aligned}$$

Inserting this formula in (B.1), using the identities $(\gamma ^0\gamma ^j)^\dagger = \gamma ^0 \gamma ^j$, $\gamma ^0 \gamma ^j \gamma ^0 \gamma ^k = - \gamma ^j \gamma ^k = \delta _{jk} + i \sigma ^{jk}$, and taking into account that $\sigma ^{jk} = -\sigma ^{kj}$, one then obtains

$$\begin{aligned} \begin{aligned} {\textrm{Im}\,}\langle \Psi , K_D\Psi \rangle&=-\frac{1}{16\pi ^2}\int _{{{\mathbb {R}}}^3} \frac{d{\varvec{p}}}{|{\varvec{p}}|}\big [|{\varvec{p}}|^2{\hat{\Psi }}_0^\dagger (1+ \nabla ^2) {\hat{\Psi }}_0 +{\hat{\Psi }}_0^\dagger \, {\varvec{p}}\cdot \nabla {\hat{\Psi }}_0 +i {\hat{\Psi }}_0^\dagger p_k\sigma ^{kj} \partial _j {\hat{\Psi }}_0\\&+|{\varvec{p}}|^2 (1+\nabla ^2){\hat{\Psi }}_0^\dagger \,{\hat{\Psi }}_0 + {\varvec{p}}\cdot \nabla {\hat{\Psi }}_0^\dagger \, {\hat{\Psi }}_0 + i \partial _j{\hat{\Psi }}_0^\dagger \sigma ^{jk} p_k {\hat{\Psi }}_0 \big ]. \end{aligned} \end{aligned}$$

Now, ${\hat{\Psi }}_0$ is a Schwarz function, and we can then integrate by parts the terms of the second line and use the elementary relations

$$\begin{aligned} \nabla ^2( |{\varvec{p}}| {\hat{\Psi }}_0)= &   \frac{2}{|{\varvec{p}}|}{\hat{\Psi }}_0+\frac{2}{|{\varvec{p}}|}{\varvec{p}}\cdot \nabla {\hat{\Psi }}_0+ |{\varvec{p}}|\nabla ^2{\hat{\Psi }}_0,\\ \nabla \cdot \left( \frac{{\varvec{p}}}{|{\varvec{p}}|}{\hat{\Psi }}_0\right)= &   \frac{2}{|{\varvec{p}}|}{\hat{\Psi }}_0+\frac{1}{|{\varvec{p}}|}{\varvec{p}}\cdot \nabla {\hat{\Psi }}_0,\\ \partial _j \left( \frac{p_k}{|{\varvec{p}}|}{\hat{\Psi }}_0\right)= &   \frac{\delta _{jk}}{|{\varvec{p}}|}{\hat{\Psi }}_0- \frac{p_k p_j}{|{\varvec{p}}|^3}{\hat{\Psi }}_0 + \frac{p_k}{|{\varvec{p}}|}\partial _j {\hat{\Psi }}_0, \end{aligned}$$

to finally obtain the statement. $\square$

### Lemma B.2

Given , with $T_{00}$ the quantum energy density of the Majorana field (4.6) there holds

$$\begin{aligned} \begin{aligned} \langle \Psi , T_{00}(0,{\varvec{x}})\Psi \rangle&= \frac{1}{2(2\pi )^6} \int _{{{\mathbb {R}}}^6} d{\varvec{p}}\, d{\varvec{q}}e^{i({\varvec{q}}-{\varvec{p}})\cdot {\varvec{x}}} \bigg \{\big (|{\varvec{p}}|+|{\varvec{q}}|\big ) {\hat{\Psi }}_0({\varvec{p}})^\dagger {\hat{\Psi }}_0({\varvec{q}})\\&\;\quad + \left( \frac{1}{|{\varvec{p}}|}+\frac{1}{|{\varvec{q}}|}\right) {\hat{\Psi }}_0({\varvec{p}})^\dagger \big [{\varvec{p}}\cdot {\varvec{q}}+i ({\varvec{p}}\wedge {\varvec{q}})\cdot \varvec{\Sigma }\big ]{\hat{\Psi }}_0({\varvec{q}})\bigg \}, \end{aligned} \end{aligned}$$

where $\Sigma _h:= \frac{1}{2} \varepsilon _{hjk}\sigma ^{jk}$. In particular $\langle \Psi , T_{00}(0, \cdot )\Psi \rangle$ is a smooth compactly supported function, whose support is contained in the one of $\Psi _0$.

### Proof

By the argument in the proof of Prop. 4.2, we can write $\Psi = \sqrt{2}a(l_{\frac{1}{2}}, l_{-\frac{1}{2}})^*\Omega$, with

$$\begin{aligned} l_{\frac{1}{2}}({\varvec{p}}):= &   l_\Phi ({\varvec{p}}) = - \frac{1}{(2\pi )^{3/2}} \nu _0({\varvec{p}})^\dagger \hat{\Phi }_0({\varvec{p}}), \\ l_{-\frac{1}{2}}({\varvec{p}}):= &   h_\Phi ({\varvec{p}}) = \frac{1}{(2\pi )^{3/2}} \hat{\Phi }_0(-{\varvec{p}})^\dagger \nu _0({\varvec{p}}), \qquad \Psi = \left[ \begin{matrix} \Phi \\ \sigma _2 \overline{\Phi }\end{matrix}\right] . \end{aligned}$$

On the other hand, from [17, Eq. (2.22)], the Majorana field can be expressed in terms of creation and annihilation operators, as a quadratic form, as

$$\begin{aligned} \psi (x)= \frac{1}{(2\pi )^{3/2}} \int _{{{\mathbb {R}}}^3} d{\varvec{p}}\bigg ( e^{ipx} \sum _{s = \pm \frac{1}{2}} u_s({\varvec{p}})a_s({\varvec{p}})^* + e^{-ipx} \sum _{s = \pm \frac{1}{2}} v_s({\varvec{p}})a_s({\varvec{p}})\bigg ), \end{aligned}$$

with 4-component spinors $u_s({\varvec{p}}), v_s({\varvec{p}})$, $s = \pm \frac{1}{2}$, defined by

$$\begin{aligned} u_{-\frac{1}{2}}({\varvec{p}})= &   \left[ \begin{matrix}\nu _0({\varvec{p}}) \\ 0\end{matrix}\right] , \quad u_{\frac{1}{2}}({\varvec{p}}) = \left[ \begin{matrix}0 \\ -\sigma _2 \overline{\nu _0({\varvec{p}})} \end{matrix}\right] , \\ v_{-\frac{1}{2}}({\varvec{p}})= &   \left[ \begin{matrix}0 \\ \sigma _2 \overline{\nu _0({\varvec{p}})} \end{matrix}\right] , \quad v_{\frac{1}{2}}({\varvec{p}}) = \left[ \begin{matrix}-\nu _0({\varvec{p}}) \\ 0\end{matrix}\right] , \end{aligned}$$

and where the creation operators $a_s^*$, $s = \pm \frac{1}{2}$, are such that $a(l,h)^* = a_{\frac{1}{2}}(l)^* + a_{-\frac{1}{2}}(h)^*$, $l, h \in L^2({{\mathbb {R}}}^3)$. It is then straightforward to compute

B.2 $$\begin{aligned} \langle \Psi ,: \psi ^\dagger (0,{\varvec{x}}) \gamma ^0\gamma ^k \psi (0,{\varvec{y}}):\Psi \rangle = \frac{2}{(2\pi )^3} \big [ v({\varvec{x}})^\dagger \gamma ^0 \gamma ^k v({\varvec{y}}) - u({\varvec{x}})^\dagger \gamma ^0 \gamma ^k u({\varvec{y}})\big ]\nonumber \\ \end{aligned}$$

with

$$\begin{aligned} u({\varvec{x}}):= &   \int _{{{\mathbb {R}}}^3} d{\varvec{p}}\, e^{-i {\varvec{p}}\cdot {\varvec{x}}} \sum _{s=\pm 1/2} \overline{l_s({\varvec{p}})} u_s({\varvec{p}}), \\ v({\varvec{x}}):= &   \int _{{{\mathbb {R}}}^3} d{\varvec{p}}\, e^{i {\varvec{p}}\cdot {\varvec{x}}} \sum _{s=\pm 1/2} l_s({\varvec{p}}) v_s({\varvec{p}}). \end{aligned}$$

Moreover, by (3.3) and (3.5), one obtains

and similarly

$$\begin{aligned} \sum _{s=\pm \frac{1}{2}} l_s({\varvec{p}}) v_s({\varvec{p}}) = - \frac{1}{2(2\pi )^{3/2}}\left( 1+\frac{p_j}{|{\varvec{p}}|}\gamma ^0 \gamma ^j\right) {\hat{\Psi }}_0({\varvec{p}}). \end{aligned}$$

Inserting these results into Eq. (B.2) and using the identity $\gamma ^0 \gamma ^j \gamma ^0 \gamma ^k =\delta _{jk} + i \sigma ^{jk} = \delta _{jk} +i \varepsilon _{jkh}\Sigma _h$, yields

$$\begin{aligned} \begin{aligned} \langle \Psi ,&: \psi ^\dagger (0,{\varvec{x}}) \gamma ^0\gamma ^k \psi (0,{\varvec{y}}):\Psi \rangle \\&=\frac{1}{(2\pi )^6} \int _{{{\mathbb {R}}}^6} d{\varvec{p}}\,d{\varvec{q}}\,e^{-i({\varvec{p}}\cdot {\varvec{x}}-{\varvec{q}}\cdot {\varvec{y}})} {\hat{\Psi }}_0({\varvec{p}})^\dagger \left( \frac{p_j}{|{\varvec{p}}|} \gamma ^0\gamma ^j\gamma ^0\gamma ^k + \frac{q_j}{|{\varvec{q}}|} \gamma ^0\gamma ^k\gamma ^0\gamma ^j\right) {\hat{\Psi }}_0({\varvec{q}})\\&=\frac{1}{(2\pi )^6} \int _{{{\mathbb {R}}}^6} d{\varvec{p}}\,d{\varvec{q}}\, e^{-i({\varvec{p}}\cdot {\varvec{x}}-{\varvec{q}}\cdot {\varvec{y}})} {\hat{\Psi }}_0({\varvec{p}})^\dagger \left[ \frac{p_k}{|{\varvec{p}}|}+\frac{q_k}{|{\varvec{q}}|}+i \varepsilon _{jkh} \left( \frac{p_j}{|{\varvec{p}}|} -\frac{q_j}{|{\varvec{q}}|}\right) \Sigma _h \right] {\hat{\Psi }}_0({\varvec{q}}) \end{aligned} \end{aligned}$$

The formula in the statement is then easily obtained from

$$\begin{aligned} \langle \Psi ,T_{00}(0,{\varvec{x}})\Psi \rangle = \frac{i}{2} \left( \frac{\partial }{\partial x_k}-\frac{\partial }{\partial y_k}\right) \langle \Psi ,:\psi ^\dagger (0,{\varvec{x}}) \gamma ^0\gamma ^k \psi (0,{\varvec{y}}):\Psi \rangle |_{{\varvec{y}}={\varvec{x}}}. \end{aligned}$$

The final part of the statement is a consequence of the fact that, from this formula, $\langle \Psi , T_{00}(0,{\varvec{x}}) \Psi \rangle$ can be expressed as a sum of terms of the form

$$\begin{aligned} (G * P_1 \Psi _0)({\varvec{x}})^\dagger P_2\Psi _0({\varvec{x}}) \quad \text {or} \quad P'_1\Psi _0({\varvec{x}})^\dagger (G'*P'_2 \Psi _0)({\varvec{x}}), \end{aligned}$$

with $G, G'$ tempered distributions and $P_j, P_j'$, $j=1,2$, differential operators. $\square$
